# Bilateral renal lymphangiectasia mimicking renal cystic disease and literature review

**DOI:** 10.1093/bjrcr/uaaf051

**Published:** 2025-10-27

**Authors:** Celal Tacyildiz, Suna Yergin Tacyildiz

**Affiliations:** Department of Radiology, Agri Training and Research Hospital, Agri 04200, Turkey; Department of Radiology, Agri Training and Research Hospital, Agri 04200, Turkey

**Keywords:** renal lymphangiectasia, cystic renal disease, perirenal cyst, hypertension

## Abstract

Bilateral renal lymphangiectasia is a rare, benign lymphatic disorder that causes the perirenal, parapelvic, and intrarenal lymphatic channels to dilate. Because it is rare and has varying radiologic appearances, it might be confused with other cystic renal illnesses, such as polycystic kidney disease, which can lead to misdiagnosis and unnecessary treatments. In this case review, a 51-year-old female patient who presented with abdominal pain was diagnosed with bilateral renal lymphangiectasia based on imaging findings. Ultrasonography, computed tomography (CT), and magnetic resonance imaging (MRI) revealed bilateral intrarenal and perirenal cysts. We comprehensively reviewed 56 cases of bilateral renal lymphangiectasia in adults published in the literature until June 2025. Conservative treatment was the preferred approach in about half of the cases. In cases where there were symptoms or complications, treatments such as percutaneous drainage, marsupialization, or nephrectomy were done. While aggressive treatment methods such as nephrectomy were commonly used in the 1980s, percutaneous interventional procedures and conservative treatments began to be used more frequently starting in the 1990s. Haematuria, ascites, hypertension, polycythaemia, pleural effusion, and renal vein thrombosis are rare complications reported. The aim of this case and literature review is to improve diagnostic accuracy by highlighting the clinical spectrum and imaging findings of bilateral renal lymphangiectasia. Accurate diagnosis based on imaging modalities is important to avoid unnecessary interventional and surgical procedures.

## Introduction

Renal lymphangiectasia is a rare, benign lymphatic disorder that causes the perirenal, parapelvic, and intrarenal lymphatic ducts to become cystic and dilate. Also known as lymphangioma or lymphangiomatosis, this condition involves mesenchymal tissues and typically presents as unilocular or multilocular cystic lesions caused by atypical dilatation of lymphatic vessels. While most lymphangiectasia are found in the neck and axillary regions, they can also occur in the retroperitoneum, mediastinum, mesentery, and pelvis.

The disease can be unilateral or bilateral, and it can be focal or diffuse. It might affect 1 or more lymphatic compartments, such as the intrarenal, parapelvic, and perirenal spaces. The underlying aetiology is unclear, but some possible mechanisms include developmental problems or secondary obstructions in the communication between the perirenal and retroperitoneal lymphatics. Familial cases have also been reported, which may imply a role for genetics.^1^

The diagnosis is made based on the content of the aspirated fluid or characteristic radiological findings. The aspiration obtained from these patients contains small amounts of protein and triglycerides and high amounts of lymphocytes. Imaging is important in diagnosing renal lymphangiectasia. Although most patients are asymptomatic, common presenting symptoms include abdominal or flank pain. Bilateral renal lymphangiectasia is very rare and may resemble other cystic kidney diseases such as polycystic kidney disease, hydronephrosis, parapelvic cysts, or cystic nephroma. It can sometimes be confused with urinoma. Treatment may vary from patient to patient depending on clinical symptoms and complications. Conservative follow-up may be performed, or cyst aspiration, sclerotherapy, marsupialization, and nephrectomy may be performed.

An accurate description of this entity is crucial to make a correct diagnosis and avoid unnecessary treatments. In this report, we describe a case of bilateral renal lymphangiectasia and comprehensively review the literature to illustrate the full range of clinical and radiologic symptoms that can occur with this rare condition.

## Case presentation

A 51-year-old woman with no known chronic disease presented to the emergency department with abdominal pain for 15 days. The lab tests showed leukocytosis (white blood cell count: 13.73 × 10^9^/L), neutrophilia (neutrophil count: 11.72 × 10^9^/L), high C-reactive protein (CRP: 39.89 mg/dL), serum creatinine 0.66 mg/dL, urea 21.5 mg/dL, sodium 137 mmol/L, potassium 3.5 mmol/L, calcium 9.66 mg/dL, and urine protein—(negative).

**Figure 1.**
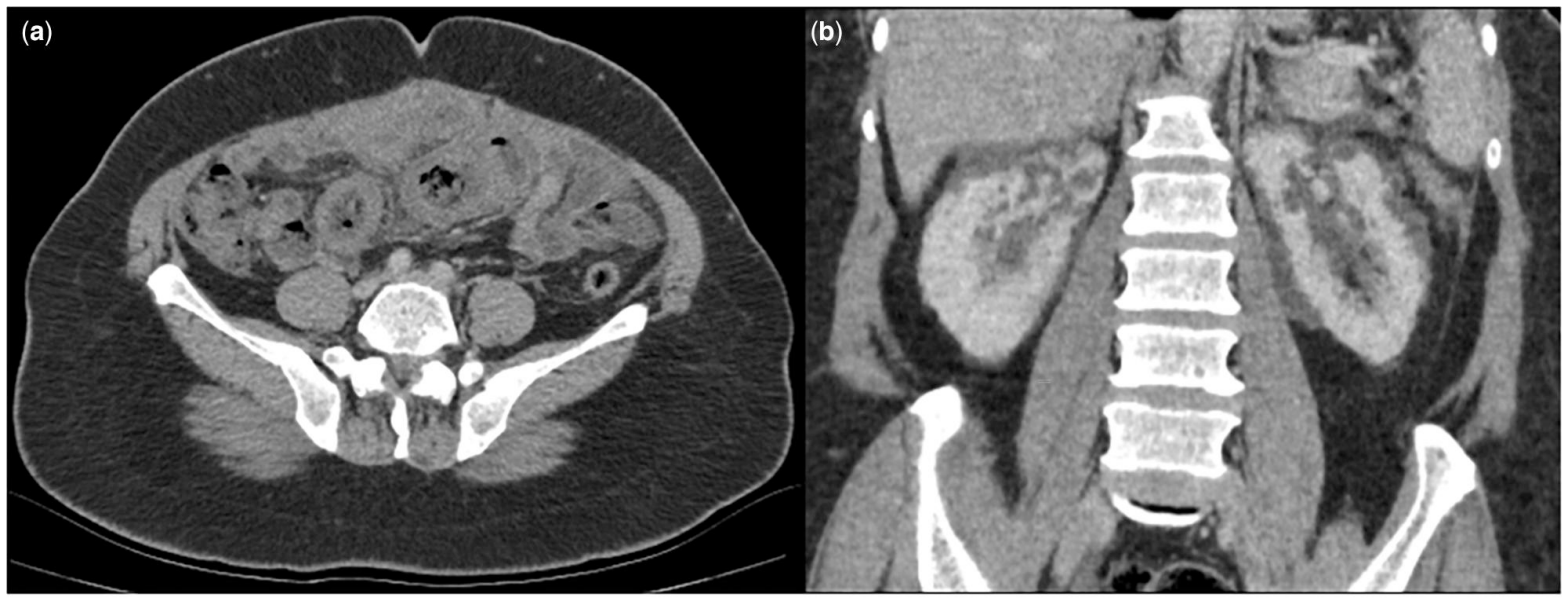
Axial and coronal contrast-enhanced abdominal CT scan demonstrates diffuse symmetric wall thickness increase in the small intestine (A) with bilateral perirenal and intrarenal cystic lesions (B).

The patient had a contrast-enhanced abdominal computed tomography (CT) scan because there were signs of infection. CT imaging showed cystic lesions up to 15 mm in diameter in both kidneys with attenuation values between 0 and 20 Hounsfield units (HU). There was also diffuse and symmetrical wall thickening of the small intestine in a long segment in the mid-abdominal region (Figure 1).

Intestinal and renal involvement of lymphoma was initially considered as differential diagnosis. However, a non-contrast abdominal CT scan performed 3.5 years ago revealed anechoic perirenal-intrarenal cysts of similar size and appearance in both kidneys. Diffuse symmetric long segmental thickening of the small intestine was absent on the previous non-contrast abdominal CT scan. The diagnosis of bilateral renal lymphangiectasia and enteritis was made because the cystic lesions in bilateral kidneys remained stable for a long time, had low HU values, and had the characteristic appearance of the cysts. Enteritis was probably associated with an infectious process and had no direct link to bilateral renal lymphangiectasia.

**Figure 2.**
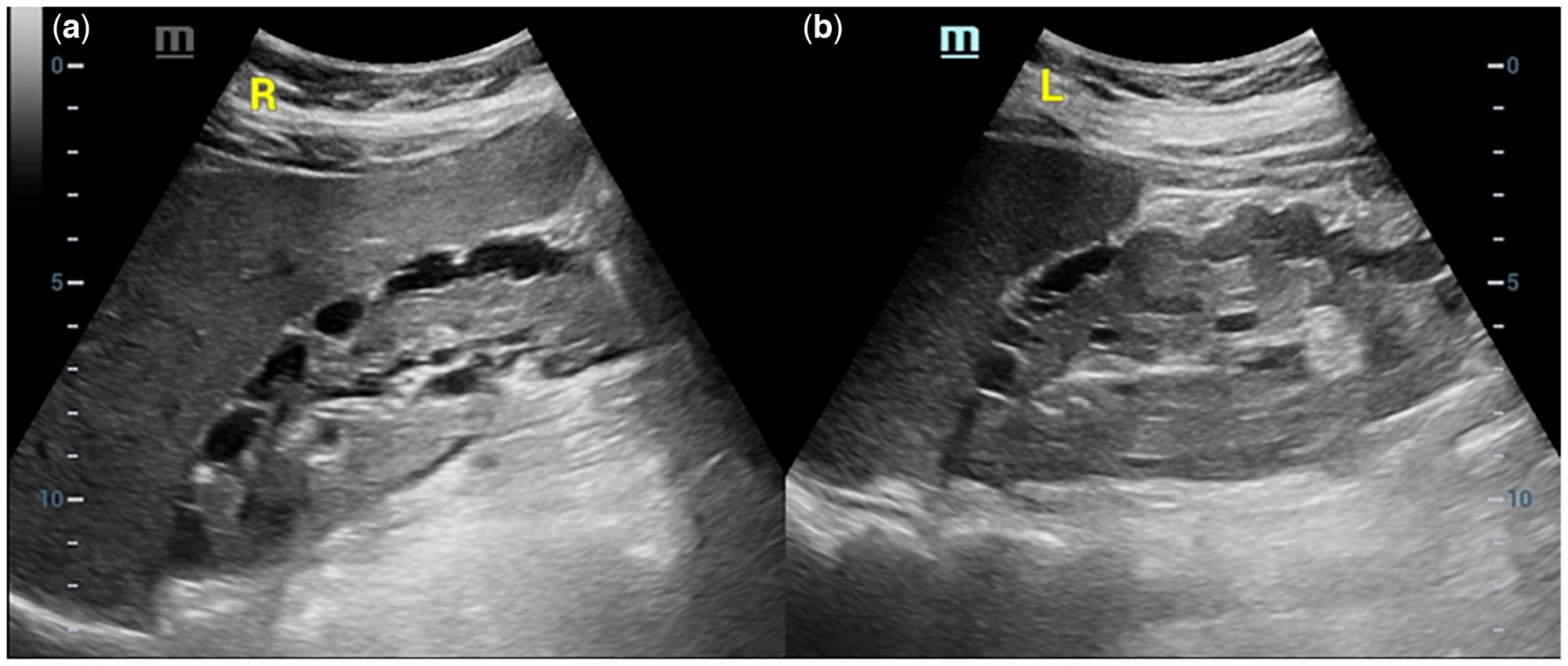
Ultrasonography of the right (A) and left kidney (B) in the longitudinal axis demonstrates bilateral perirenal and intrarenal cystic lesions.

**Figure 3.**
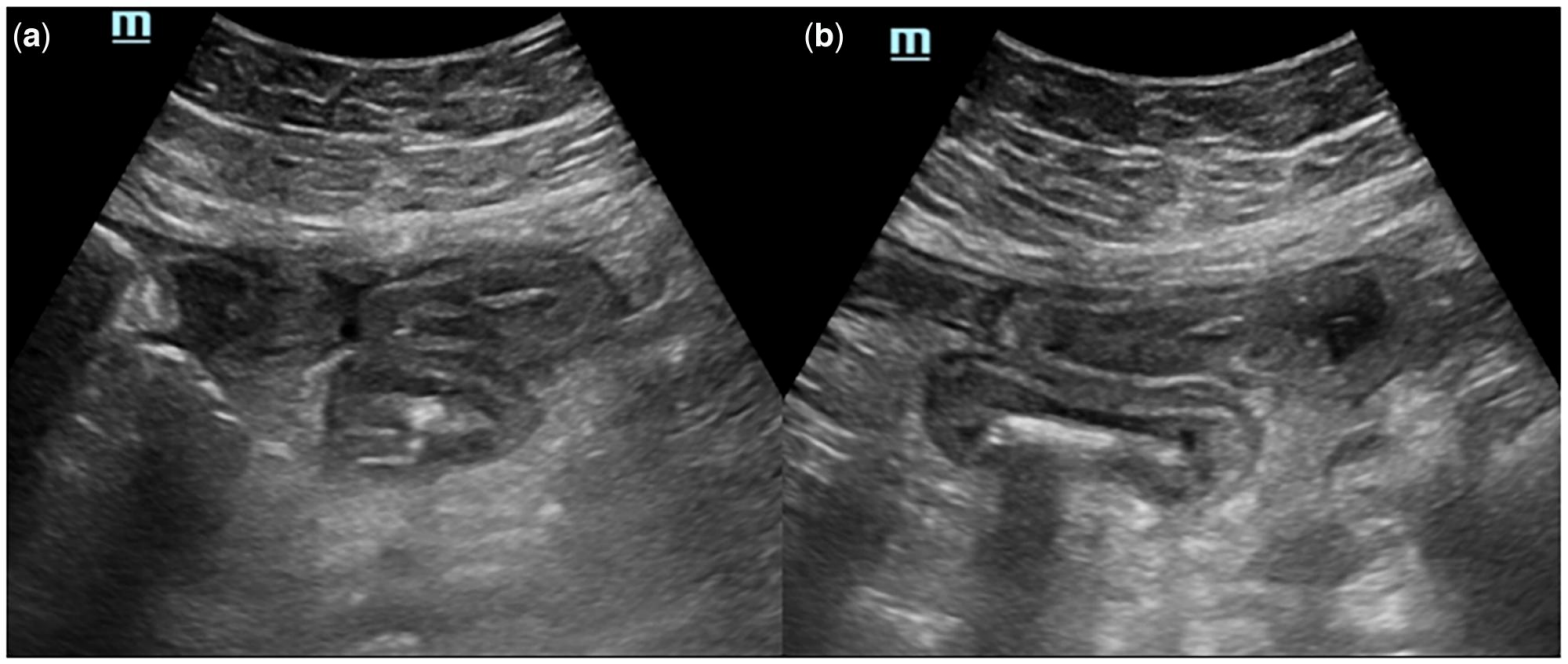
Abdominal ultrasonography demonstrates diffuse and symmetrical wall thickening of the small bowel loops in a long section of the abdomen at midline level (A, B).

Abdominal ultrasonography performed 1 day later showed multiple thin-walled, anechoic perirenal-intrarenal cysts in both kidneys. Abdominal ultrasonography also showed diffuse symmetric long segmental thickening of the bowel wall (Figures 2 and 3).

**Figure 4.**
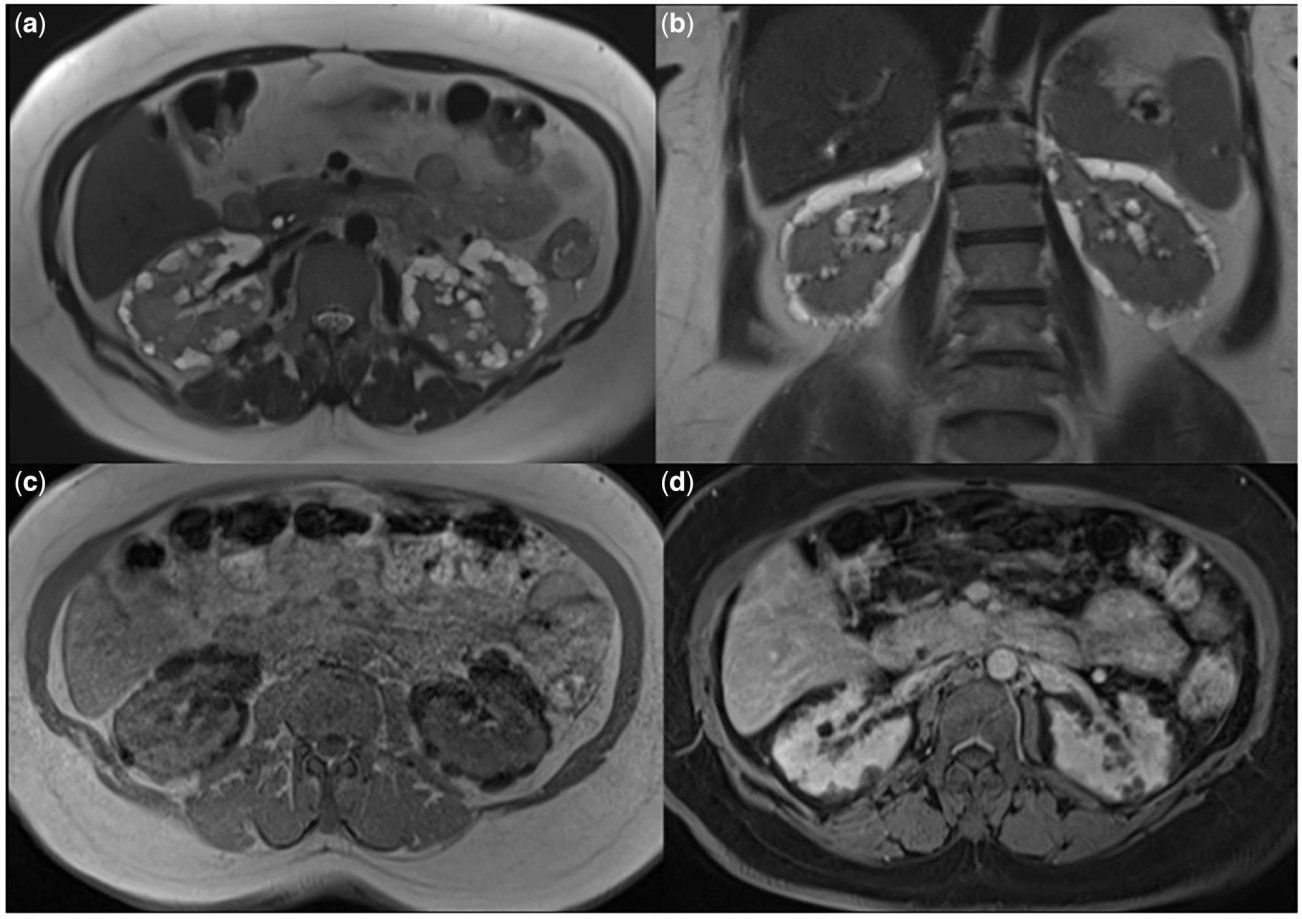
Bilateral perirenal and intrarenal cystic lesions are hyperintense on axial (A) and coronal (B) T2 MR images and hypointense on axial (C) T1 MR images. Contrast enhancement was not seen in bilateral perirenal and intrarenal cystic lesions on axial fat-suppressed contrast enhanced (D) T1 images.

**Figure 5.**
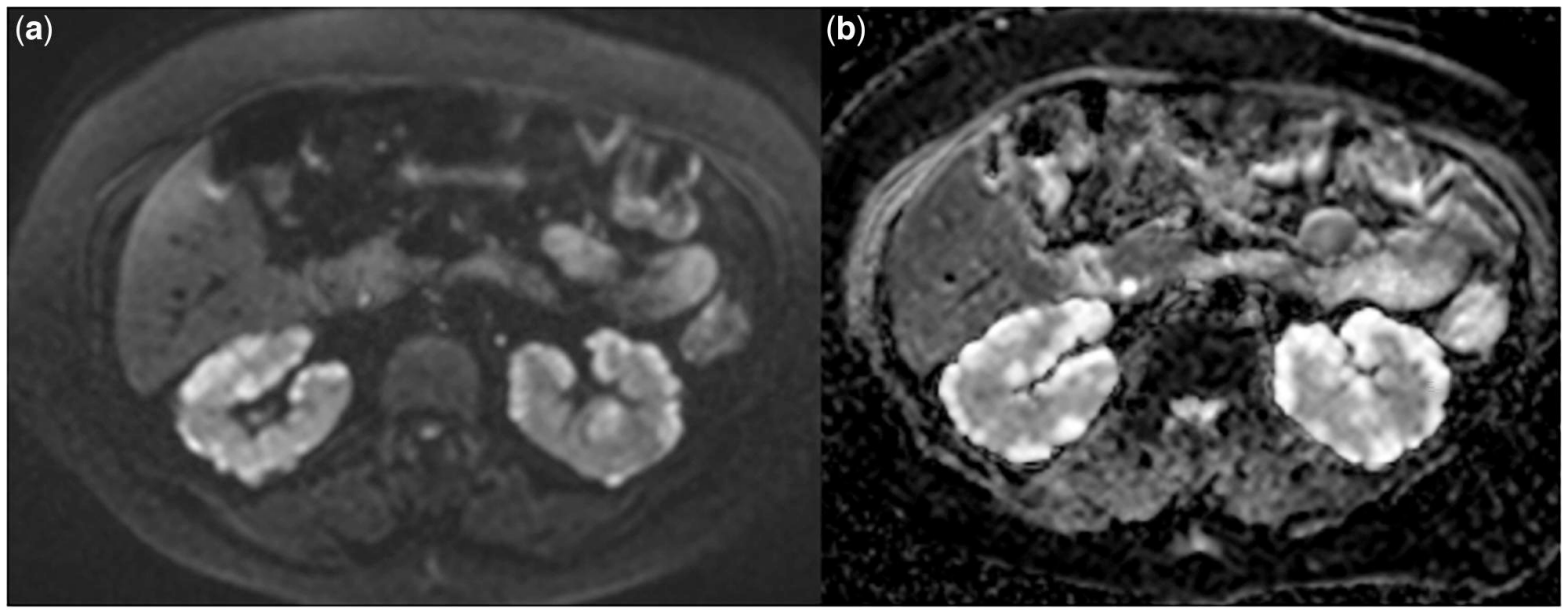
Bilateral perirenal and intrarenal cystic lesions are hyperintense on DWI (A) and ADC maps (B).

Thereafter, contrast-enhanced magnetic resonance imaging (MRI) showed perirenal cysts that were hypointense on T1-weighted images and hyperintense on T2-weighted images. The cysts did not show contrast enhancement on MRI (Figure 4). DWI showed no diffusion restriction in bilateral perirenal cysts (Figure 5).

Since our patient did not have any complications such as hypertension, haematuria, ascites, renal vein thrombosis, or renal failure, she was treated conservatively with regular follow-ups for bilateral renal lymphangiectasia. Antibiotherapy was given for enteritis. After 2 weeks, the patient’s complaint of abdominal pain regressed, and laboratory values returned to normal.

## Literature review

Until June 2025, our review of the literature revealed 56 cases of bilateral renal lymphangiectasia in adults. Including our case, the total number of documented cases has increased to 57. Among these patients, there were 29 females and 28 males, suggesting no significant difference between the sexes. The mean age at the time of diagnosis was 39.8 years (20-74) (Table 1).

**Table 1.** Demographic, clinical, histological characteristics, and treatment of adult patients with bilateral renal lymphangiectasia.

Case no	Publication	Year of publication	Age/sex	Clinical presentation	Histology cytology	Treatment
1	De Maeyer et al^14^	1982	43/M	Flank pain	Endothelium-lined thin-walled lymphatic spaces	Resection (lumbotomy)
2	Murakumo et al^15^	1986	37/F	Abdominal pain	Endothelium-lined thin-walled lymphatic spaces	Resection
3	Kutcher et al^16^	1987	74/F	Abdominal pain	Cysts lined by endothelial cells (F VIII)	Nephrectomy
4	Meredith et al^1^	1988	23/F	Abdominal pain	Endothelium-lined thin-walled lymphatic spaces	Nephrectomy
5	Meredith et al^1^	1988	NA/F	Abdominal pain	NA	Conservative
6	Murray et al^17^	1991	30/M	Flank pain	Endothelium-lined thin-walled lymphatic spaces	Nephrectomy
7	Schwarz et al^13^	1993	42/M	Abdominal pain, distention, hypertension	Lymphatic fluid, renin	Conservative resection (marsupialization)
8	Burton et al^4^	1994	35/M	Hypertension	Lymphatic fluid, leucocytes	Percutaneous drainage
9	Leder et al^18^	1995	NA/F	Abdominal distention	NA	NA
10	Riehl et al^9^	1997	22/M	Flank pain, hematuria, renal vein thrombosis	Lymphatic fluid	Conservative
11	Varela et al^19^	1997	50/F	Abdominal pain	Endothelium-lined thin-walled lymphatic spaces	NA
12	Ozmen et al^2^	2001	35/F	Flank pain, hematuria, hypertension	Lymphatic fluid	Percutaneous drainage
13	Ramseyer^20^	2001	28/M	Hematuria	NA	NA
14	Llorente et al^21^	2002	30/M	Hematuria	Lymphatic fluid, lymphocytes	Conservative
15	Shaheen et al^3^	2003	36/M	Fatigue, facial swelling	Thin-walled lymphatic spaces	Conservative resection (marsupialization)
16	Kocaoglu et al^22^	2005	20/M	Hypertension	NA	NA
17	Sarikaya et al^23^	2006	53/F	Flank pain	NA	Percutaneous drainage
18	Pianezza et al^24^	2006	52/M	Flank pain, Hematuria	NA	Conservative
19	Ashraf et al^25^	2007	23/F	Abdominal pain, hypertension	Lymphatic fluid, lymphocytes	Conservative
20	Rastogi et al^26^	2008	20/M	Abdominal pain, fever	Lymphatic fluid	Percutaneous drainage
21	Navarro-Sanchis et al^27^	2008	66/M	Hypertension	NA	NA
22	Chen et al^28^	2009	34/F	Flank pain, fever	Lymphatic fluid	Nephrectomy
23	Bagheri et al^29^	2009	21/M	Abdominal pain, dyspepsia	NA	NA
24	Ali Al-Dofri^30^	2009	22/M	Abdominal pain, distention	Lymphocytes	Percutaneous drainage
25	Antonopoulos et al^31^	2010	39/F	Abdominal pain	Lymphatic fluid	Conservative
26	Antonopoulos et al^31^	2010	37/M	Hypertension	NA	Conservative
27	Rastogi et al^32^	2010	60/M	Abdominal pain, distention	Lymphocytes, renin	Conservative
28	Bazari et al^7^	2010	49/M	Flank pain	NA	Conservative resection (marsupialization)
29	Magu et al^33^	2010	28/M	Abdominal pain	Lymphatic fluid	Percutaneous drainage
30	Hakeem et al^34^	2010	50/F	Urine retention, uterine prolapse	Lymphatic fluid, lymphocytes	Conservative
31	Wani et al^35^	2011	60/M	Fatigue	Lymphocytes	Conservative
32	Viglietti et al^6^	2012	49/M	Renal insufficiency	Lymphatic fluid	Percutaneous drainage
33	Vasconcelos et al^36^	2012	65/M	Dyspnoea	NA	Conservative
34	Karkouche et al^37^	2013	22/F	Flank pain	Cysts lined by endothelial cells	Conservative resection (marsupialization)
35	Kumar et al^38^	2014	32/F	Flank pain, hypertension	Lymphocytes, renin	Conservative
36	Blanc et al^5^	2014	58/M	Hypertension, polycythemia	Lymphocytes	Conservative resection (marsupialization)
37	Nassiri et al^39^	2015	24/M	Abdominal distention, dyspnoea	Lymphatic fluid	Conservative
38	Elbanna et al^40^	2015	38/M	Flank pain	NA	Conservative
39	Bansal et al^41^	2016	40/F	Flank pain	NA	Conservative
40	Anwar et al^42^	2016	42/M	Abdominal pain, hematuria	Lymphatic fluid	NA
41	Renacci et al^43^	2017	30/M	Chronic right hip pain	NA	Conservative
42	Pandya et al^44^	2017	34/M	NA	Lymphatic fluid, lymphocytes	Conservative
43	Pandya et al^44^	2017	28/M	Flank pain	Lymphocytes	Conservative
44	Pandya et al^44^	2017	40/F	Flank pain, hypertension	NA	Conservative
45	Pandya et al^44^	2017	62/F	NA	NA	Conservative
46	Umapathy et al^11^	2020	69/F	Abdominal pain	NA	Conservative
47	Umapathy et al^11^	2020	43/M	Flank pain, abdominal distention	Lymphatic fluid	Conservative
48	Guadagni et al^45^	2020	27/F	Abdominal pain	NA	Conservative
49	Alzahrani et al^46^	2021	39/F	Abdominal pain	NA	Conservative
50	Jokar et al^8^	2021	38/M	Flank pain	NA	Conservative
51	Cerba et al^47^	2022	34/F	Flank pain, hypertension	Lymphatic fluid	Conservative
52	Bouardi et al^48^	2023	24/F	Blunt abdominal trauma	Lymphatic fluid	Percutaneous drainage
53	Ayed et al^49^	2024	55/F	Flank pain	NA	Conservative
54	Venkatachalam et al^50^	2024	34/F	Flank pain, hypertension	Endothelium-lined thin-walled lymphatic spaces	Conservative resection
55	Peñaherrera-Vásquez et al^51^	2025	55/M	Flank pain, fever	Lymphatic fluid, lymphocytes	Conservative
56	Chirayath et al^12^	2025	36/F	Flank pain,	Lymphatic fluid	Conservative resection (marsupialization)
57	Present case	2025	51/F	Abdominal pain	NA	Conservative

Abbreviations: F = female; M = male; NA = not available.

About half of the patients had abdominal or flank pain (Table 2). The diagnosis is usually based on the typical imaging findings of the disease. The protein content of the lymphatic fluid, along with a biochemical analysis that shows lymphocytes, can sometimes help confirm the diagnosis. Histopathologic or cytologic evidence supported the diagnosis in 35 cases of bilateral renal lymphangiectasia, while imaging findings were the only evidence in 22 cases (Table 1).

**Table 2.** Summary of presenting symptoms in literature of adult patients with bilateral renal lymphangiectasia.

Clinical presentation	Count	Percentage (%)
Abdominal/flank pain	39	50.6
Hypertension	12	15.6
Hematuria	6	7.8
Abdominal distention	6	7.8
Fever	3	3.9
Dyspnoea	2	2.6
NA	2	2.6
Fatigue	2	2.6
Renal vein thrombosis	1	1.3
Renal insufficiency	1	1.3
Other	3	3.9

**Table 3.** Summary of treatment in literature of adult patients with bilateral renal lymphangiectasia.

Treatment	Count	Percentage (%)
Conservative	29	50.9
Percutaneous drainage	8	14
NA	7	12.3
Conservative resection (marsupialization)	6	10.5
Nephrectomy	4	7
Resection/conservative resection	3	5.3

Approximately half of the cases reported in the literature were treated conservatively (Table 3). Hypertension, ascites, haematuria, polycythaemia, pleural effusion, and renal vein thrombosis have been reported, but these are rare. When such problems occur, patients undergo percutaneous drainage, conservative resection (marsupialization), drug therapy, and sometimes nephrectomy. While aggressive treatment methods such as nephrectomy were commonly used in the 1980s, percutaneous interventional procedures and conservative treatments began to be used more frequently starting in the 1990s. This improvement may be due to advances in imaging techniques and increased awareness of the disease, which have led to an increased likelihood of accurate diagnosis, as well as developments in percutaneous treatment methods.

There have been reports of cases getting worse during pregnancy and needing procedures like percutaneous drainage and surgery.^1^^,^^2^ These patients recovered after percutaneous drainage and surgery. After pregnancy, the size of the perirenal fluid collection did not change during follow-up. The finding suggests that the disease may worsen during pregnancy in some individuals, and treatment may be needed. In 2 cases described in the literature, this condition led to preterm delivery.^1^^,^^2^

In some patients with renal lymphangiectasia, erythropoietin and renin hormones were found in the fluid obtained during an interventional procedure. The authors said that polycythaemia occurred secondary to the presence of the erythropoietin hormone.^3–8^

Thrombus in the renal vein was reported in a patient with bilateral renal lymphangiectasia.^9^ There are different opinions in the literature regarding the relationship between renal vein thrombosis and renal lymphangiectasia.

Villard et al^10^ and Umapathy et al^11^ reported that renal vein thrombosis causes impaired lymphatic drainage leading to the development of lymphangiectasia. Riehl et al^9^ reported thrombus in the renal vein secondary to the compression of cysts in renal lymphangiectasia. There is a need for detailed studies on this subject with more case groups.

Cysts in renal lymphangiectasia rarely can significantly compress the kidney and cause page kidney.^12^^,^^13^ In this case, the patient may need surgery to remove the cyst that is compressing the kidney.

## Discussion

Our case adds to the small number of reported bilateral renal lymphangiectasia cases. Several systemic or neoplastic conditions, such as lymphoma, Erdheim-Chester, and liposarcoma, may also present with bilateral renal involvement in adult patients. Perirenal renal lymphangiectasia may be confused with these diseases. However, radiologists play an important role in differential diagnosis. This is because ultrasound, CT, and MRI reveal characteristic findings that are essential for diagnosis. Particularly, perirenal renal lymphangiectasia exhibits anechoic cysts, while lymphoma and Erdheim-Chester disease display soft tissues. In lymphomas and Erdheim-Chester diseases, soft tissues show internal vascularization on Doppler ultrasonography. On CT scans, the density of these soft tissues is above 20 HU. These features allow differentiation between cysts and soft tissues located around the kidney.

In hydronephrosis, CT and MRI show contrast material in the collecting system in the urography phase, whereas in renal lymphangiectasia, contrast filling is not seen in the urography phase. In adult polycystic kidney disease, there are cysts of various sizes and scattered locations. These cysts are accompanied by liver and pancreatic cysts. Lymphatic scintigraphy may be used in the diagnosis of renal lymphangiectasia, although it is not often preferred. In conclusion, a significant number of cases in the literature show that imaging alone can often be used to make a diagnosis without the need for histologic confirmation.

Once diagnosed, it is important to keep the patient’s blood pressure and renal function tests under observation for a long period of time for conditions such as hypertension, ascites, haematuria, and rarely renal failure.

## Conclusion

Bilateral renal lymphangiectasia is an extremely rare pathology and rarely leads to complications. In case of complications, percutaneous interventional procedures and conservative resection are needed. Radiologic recognition of this rare condition prevents misdiagnosis of cystic kidney disease and unnecessary surgery in asymptomatic cases.

## Learning points

- Bilateral renal lymphangiectasia is a rare benign condition that mimics other cystic renal diseases.
- Accurate diagnosis via US, CT, and MRI avoids unnecessary interventions or surgeries.
- Pregnancy may worsen symptoms, indicating a need for closer monitoring in such patients.
- Rare complications include hypertension, polycythaemia, renal vein thrombosis, and ascites.
